# Socioeconomic Disparities in Breast Cancer Survival: Examining Potential Mediator Role of Oncotype DX(ODX) Test and Stage at Diagnosis Among HR+/HER2- Breast Cancer Women

**DOI:** 10.3390/cancers17111802

**Published:** 2025-05-28

**Authors:** Pratibha Shrestha, Qingzhao Yu, Edward S. Peters, Edward Trapido, Mei-Chin Hsieh, Tekeda Ferguson, Quyen D. Chu, Xiao-Cheng Wu

**Affiliations:** 1 Division of Population Health Sciences, Department of Surgery, College of Medicine, University of Florida, Gainesville, FL 32610, USA; 2 Louisiana Tumor Registry, Epidemiology Program, School of Public Health at LSU Health New Orleans, New Orleans, LA 70112, USA; 3 Biostatistics Program, School of Public Health at LSU Health Sciences Center, New Orleans, LA 70112, USA; 4 Department of Epidemiology, College of Public Health, University of Nebraska Medical Center, Omaha, NE 68198, USA; 5 Surgical Oncology, Howard University Hospital, Washington, DC 20060, USA

**Keywords:** socioeconomic status, Oncotype DX, stage, breast cancer, survival

## Abstract

Background: Women with a lower socioeconomic status (SES) have an increased risk of dying from breast cancer (BC) than those with a higher SES. The association of SES with BC survival may be partially mediated by factors such as Oncotype DX (ODX) testing and stage at diagnosis. This study aims to examine SES disparities in survival among HR+/HER2- BC women and to quantify the mediating effects of the ODX test and stage. Methods: We used data from the Louisiana Tumor Registry to identify women aged 20–90 years diagnosed with stage I–II in 2011–2014 and stage I–III in 2015–2017 HR+/HER2- BC who underwent BC surgery. The follow-up cutoff date was 31 December 2020. Cox proportional hazard regression and generalized mediation analysis were both performed. Results: Of 8931 BC women, 41.4% underwent ODX testing. After adjusting for sociodemographic, tumor characteristic, and treatment variables, low SES women had a higher hazard of overall death (HR = 1.16, 95% CI: 1.02–1.32) and BC-specific death (HR = 1.37; 95% CI: 1.01–1.87) compared to high SES women. The ODX test and stage explained 9.0% and 11.2% SES differences in the hazard of overall death and 4.4% and 13.3% in BC-specific death, respectively. Conclusions: Low SES is associated with higher hazard rates of overall and cause-specific death among women with breast cancer, even after adjustment. Differences in Oncotype DX (ODX) testing and stage at diagnosis explained part of these disparities. Targeted interventions are needed to improve access to genomic testing and early detection to reduce SES-related disparities in breast cancer outcomes.

## Simple Summary

Those with a lower socioeconomic status (SES) face a higher risk of dying from breast cancer compared to those with a higher SES. This study aims to understand how SES affects survival rates among women with hormone receptor-positive, HER2-negative breast cancer. By examining data from the Louisiana Tumor Registry, we found that SES disparities in survival are partly due to differences in Oncotype DX testing and the stage at diagnosis. Our findings suggest that improving access to these tests and early detection can help reduce the risk of death for women with low SES. This research highlights the need for targeted interventions to improve breast cancer outcomes for disadvantaged groups.

## 1. Introduction

### 1.1. Background

In 2024, it was estimated that there were 310,720 new cases of breast cancer (BC) and approximately 42,250 deaths from BC in the United States (U.S.) [1]. The 5-year and 10-year relative survival rates for women with invasive BC are 90% and 84%, respectively [2]. Notably, the 5-year relative survival has improved from 76% in 1975–1977 to 92% in 2009–2015 for White women, while for Black women it has improved from 62% to 83% [3]. Despite substantial improvements in BC survival rates over time, socioeconomic status (SES) disparities in survival persist [3]. Research has shown that women with a lower SES had a 43% higher risk of dying from BC than those with a higher SES [4].

### 1.2. Importance of the Study

Understanding the impact of SES on breast cancer outcomes is crucial for developing targeted interventions to reduce health disparities. This study investigates the role of Oncotype DX (ODX) testing and stage at diagnosis in mediating SES disparities in survival among women with early-stage breast cancer. By examining these factors, this research aims to provide insights that can inform clinical practice and policy decisions, ultimately promoting equity in healthcare access and treatment.

### 1.3. SES, Genomic Testing, and Stage

The persistent SES disparities in survival may be attributable to multiple factors, including Oncotype DX (ODX) testing (a multigene marker test) [5] and stage at diagnosis (defined by the extent and spread of cancer) [4,6,7] in the causal pathways. ODX is a genomic test that predicts the likelihood of chemotherapy benefit [5] and the 10-year risk of distant recurrence [8,9] for early-stage BC (ESBC) women with hormone receptors positive/human epidermal growth factor 2—neu negative (HR+/HER2-). It is a valuable tool in the decision-making process for adjuvant treatment as it provides personalized information about chemotherapy and identifies patients with high-risk recurrence scores [10,11] who may benefit from the chemotherapy [12,13,14] while avoiding unnecessary toxicity in low-risk patients [9,15]. Studies found that patients who received the ODX test tended to have improved survival rates [16,17] and reduced chemotherapy toxicity [18]. Prior research also demonstrated that low SES is associated with lower ODX testing [19], in addition to a higher stage at BC diagnosis [7,20] and poorer survival rates [4,6]. Therefore, the ODX test and stage at diagnosis may partially explain SES disparities in survival.

The National Comprehensive Cancer Network (NCCN) 2021 and the American Society for Clinical Oncology (ASCO) 2007 guidelines recommended an ODX test to assess the chemotherapy benefit for HR+/HER2- patients with lymph-node negative (pN0) or 1–3 positive lymph-nodes (pN1) BC [21,22]. HR+/HER2- is the most common subtype of BC, which has the best prognosis; HR+ refers to the presence of estrogen and/or progesterone receptors in the tumor cells, and HER2- refers to the presence of a high level of a protein called HER2/neu [23]. However, women with HR+/HER2- BC may not always benefit from or require adjuvant chemotherapy as women at low risk of disease recurrence can be treated with endocrine therapy alone [24,25]. In addition to the recommended use in certain patients, the results obtained from ODX testing directly affect the treatment decision of patients and their doctors based on their recurrence score (RS) [5,26], ultimately affecting their survival. Despite NCNN guidelines recommendations and the survival benefits, the ODX test is underutilized [27]. Women residing in low SES census tracts were less likely to do the test, with the proportion increasing with higher SES categories [27,28]. The need for adherence to guideline-concordant care, especially for women with low SES, needs to be highlighted. To our knowledge, no prior published research examines the role of ODX test receipt as a potential mediator between SES and survival. Moreover, previous studies [6,29] explored only a few aspects of SES, such as income, education, insurance, and federal poverty level. They often did not use the SES composite score based on Yost et al. [30], which includes seven elements of SES (median household income, median house value, median rent, percent below 150% of the poverty line, education index, percent working class, and percent unemployed) measured at the census tract.

### 1.4. Research Aims

**Figure 1:**
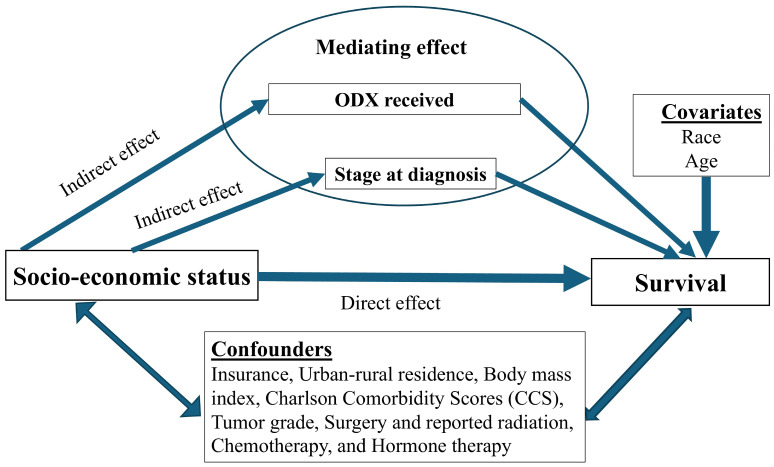
Conceptual model.

Our study aims to examine neighborhood SES disparities in BC survival among HR+HER2- BC women and to quantify the mediating effects of ODX use and stage at diagnosis on the association of SES with BC survival (Figure 1). By adjusting for treatment variables, we aim to isolate the pathway between SES and survival through ODX use and stage, while controlling for other covariates.

## 2. Methods and Material

### 2.1. Data Source and Study Design

Data were obtained from the population-based Louisiana Tumor Registry (LTR) [31]. The eligible criteria included Louisiana women aged 20–90 years, diagnosed with microscopically confirmed invasive ESBC between 2011 and 2017 [32], who underwent either lumpectomy or mastectomy. The HR+/HER2- subtype consists of tumors with estrogen receptor (ER-positive or borderline) and progesterone receptor (PR-positive or borderline) [33,34], and negative or borderline HER2 status [35].

**Figure 2:**
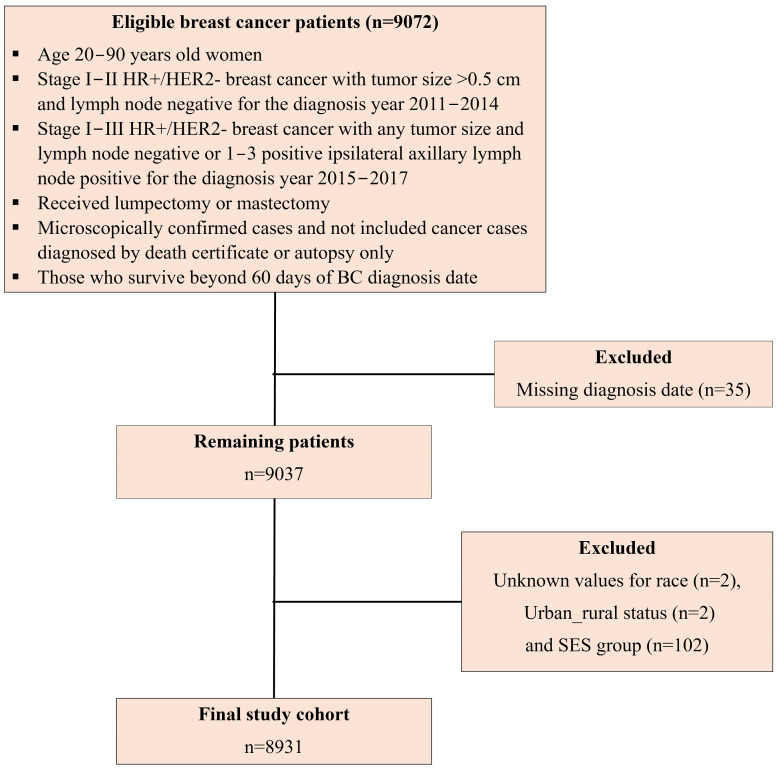
Study flow diagram for patient’s inclusion and exclusion criteria.

To align with the changes in NCCN Clinical Practice Guidelines for ODX testing over time, we restricted our data to stage I-II HR+/HER2- breast cancer cases with tumor size > 0.5 cm that were lymph node-negative for the diagnosis years 2011–2014. For diagnosis years 2015–2017, we included stage I–III HR+/HER2- breast cancer with any tumor size that were lymph node-negative or 1–3 positive ipsilateral axillary lymph node-positive [19]. We excluded cases identified only through death certificates or autopsies, as well as those who died within 60 days of the BC diagnosis, to ensure adequate time for ODX testing and treatment initiation (Figure 2). Additionally, we excluded cases with missing diagnosis dates, and unknown values for race, urban–rural status, and SES group.

This study was reviewed and approved by Louisiana State University Health Science Center Institutional Review Board IRB#2368 on 18 March 2022.

### 2.2. Outcome

Overall survival (OS) and BC-specific survival were the two outcome variables. The study endpoint date was 31 December 2020. LTR linked its cancer data to the state death to add certificate and US national death index data up yearly to 2020. Survival time was estimated from the date of BC diagnosis to the endpoints (death or life).

### 2.3. Exposure

This study contributes to the existing literature by using the Yost SES index, a composite measure based on median household income, median house value, median rent, percent below 150% of the poverty line, education index, percent working class, and percent unemployed [30], all derived from the American Community Survey (ACS) 5-year estimates. The ACS (2010–2014) and ACS (2013–2017) estimates were used for BC diagnosed in 2011–2014 and 2015–2017, respectively [30]. This refined approach enhances the classification of SES, allowing for a more comprehensive understanding of how socioeconomic status influences BC outcomes, unlike previous studies [6,29] focusing on a few indicators of SES.

The exposure variable was low SES or high SES. Each patient was categorized into the low SES group if they fell within the 1st, 2nd, and 3rd SES quintiles, as preliminary analysis showed these quintiles had a higher hazard of deaths. Patients in the 4th and 5th SES quintiles were categorized into the high SES group, using the available census tract-based SES quintiles from SEER incidence and survival databases.

### 2.4. Mediator

Mediators are variables that can explain the pathway through which exposure affects the outcome. The criteria to be mediator is (1) the independent variable (exposure) significantly predicts the mediator, and (2) the mediator significantly predicts the dependent variable (outcome) [36,37]. In other words, the mediator lies on the causal pathway between the exposure and the outcome. Our primary mediator of interest was ODX use (yes or no). Patients who underwent ODX testing within 12 months after cancer diagnosis and had received the test results were categorized into the ODX Yes group. We verified ODX testing by confirming the availability of the recurrence score (RS). The second mediator of interest was the stage at diagnosis. The AJCC (American Joint Committee on Cancer) 7th edition [38] was used for stage classification. It was categorized into stages I–III.

### 2.5. Covariates/Confounders

Covariates included race (white, black, or other), age at diagnosis (<50, 50–59, 60–69, 70–79, and ≥80 years), insurance status at diagnosis (not insured, Medicaid, Medicare, other public health insurance: Tricare, Military, Veterans Affairs, and Indian/Public Health Service, and unknown), and urban–rural residence representing the percent of the population living in an urban area at census tract level (all urban 100%, mostly urban ≥50%–<100%, mostly rural >0–<50%, and all rural 0%) [39], BMI was calculated in kg/m^2^ and classified as underweight (<18.5), normal weight (18.5–<25), overweight (25–<30), and obesity (≥30) [40], and Charlson/Deyo Comorbidity Scores (CCS: based on 16 comorbidities and classified as 0, 1, and 2+ comorbidity scores) [41]. Tumor grade was classified as grade 1 (well-differentiated), grade 2 (moderately differentiated), grade 3/4 (poorly differentiated/undifferentiated) [42], and grade unknown. Other covariates included surgery and reported radiation (lumpectomy plus radiation, mastectomy plus radiation, lumpectomy with no/unknown radiation, and mastectomy with no/unknown radiation), and reported chemotherapy (yes, no, or unknown) and hormone therapy (yes, no, or unknown).

Covariates were selected based on existing literature and using directed acyclic graphs (DAGs) to ensure comprehensive coverage of relevant factors influencing BC survival [43,44].

### 2.6. Statistical Analysis

Chi-square tests were conducted by SES and ODX testing to observe the differences in the distribution of each covariate between patients with low and high SES, as well as between patients with an ODX test and those without. The significance level was set at *p* = 0.05. Cox proportional hazards regression was performed to calculate the hazard ratios (HRs) and 95% confidence intervals (CIs) for overall and BC-specific survival (BCSS). The proportionality hazard (PH) assumption was tested using the proportionality test and checked cumulative martingale residuals (supremum test). Multicollinearity among covariates was evaluated using variance inflation factors (VIF), and VIF > 5 indicates multicollinearity. The crude model examines bivariate relationships between each predictor and survival outcome, allowing us to assess unadjusted associations before proceeding to multivariate modeling.

The multiple mediation analysis of survival data was performed utilizing the method described by Yu et al. [36,45] using mma package in R. To determine ODX use and stage at diagnosis as potential mediators, we first tested the significance of two associations as a prior examination. These screening tests were (1) between SES and each potential mediator (ODX use and stage), and (2) between each potential mediator (ODX use and stage) and the survival outcome, adjusting for all other variables controlled in the model. The significance level for both conditions was set at 0.05. ODX use and stage at diagnosis were selected as potential mediators since both associations mentioned above were significant. We performed the generalized mediation analysis using the ‘mma’ function in R [36,45] setting the bootstrap iterations number as 10,000 to differentiate effects from SES to BC survival through ODX testing and stage at diagnosis. All data analyses were performed using SAS 9.4 and R 4.2.0.

## 3. Results

Of the 8931 women diagnosed in 2011–2017 with early-stage (I–III) HR+/HER2- BC, 52.2% were in the low SES group. The majority of Black patients (78.55%), Medicaid insured patients (74.23%), uninsured patients (69.70%), those residing in rural areas (80.48%), obese patients (56.30%), and those with ≥2 comorbidities (64.63%) belonged to the low SES group. (Table 1) The highest distribution of stage III diagnoses (59.82%) and higher tumor grades III/IV (58.20%) were observed in the low SES group. Of the total cohort, 44.45% (3970 women) underwent ODX testing while 55.55% (4961 women) did not. Among those who received ODX testing, the distribution was relatively balanced between SES groups (49.72% low SES, 50.28% high SES). (Table 1) However, among those who did not receive ODX testing, there was a higher proportion of women from low SES areas (54.20%) compared to high SES areas (45.80%) Among the 24% patients who received chemotherapy, 55.33% were from low SES and 44.67% were from high SES. Among all 1160 deceased patients, 17.84% died due to BC, while the remaining cases died from other causes. Of those who died from BC, 64.25% belonged to the low SES group, while 35.75% belonged to the high SES group.

The differences between ODX recipients and non-ODX recipients were significantly associated with SES, race/ethnicity, age at diagnosis, insurance status, and tumor characteristics (Table 2). The proportion of ODX testing was higher among White patients than Black patients (45.74% vs. 40.14%, *p* < 0.0001) and was notably higher in the 50–59 age group (53.52%, *p* < 0.0001) compared to other age groups. Patients with private insurance had a higher proportion of ODX receipt compared to those with Medicare (49.75% vs. 40.00%, *p* < 0.0001). Additionally, AJCC stage I patients who received ODX testing made up a larger proportion compared to higher stages (46.41%, *p* < 0.0001), and those with grade II tumors were also more likely to receive ODX testing (47.22%, *p* < 0.0001). Furthermore, ODX testing was more frequent among patients who underwent lumpectomy plus radiation (49.23%, *p* < 0.0001) compared to other surgery and reported radiation categories, and those receiving hormone therapy (47.39%, *p* < 0.0001) compared to those who did not receive hormone therapy (Table 2).

### 3.1. Factors Associated with OS and BCSS for Women with Stage I-II Breast Cancer

The median follow-up time for the entire cohort was about 62.50 months. The proportionality assumption was met for SES in OS (*p* = 0.72) and BCSS (*p* = 0.19). There was no severe (VIF < 5) multicollinearity observed among the covariates. Table 3 demonstrates factors associated with OS. In the crude model, women in areas with low SES have a higher risk of dying from all causes than women in areas with high SES (HR = 1.45; 95% CI: 1.29–1.63). After adjustment for the other covariates, the hazard of death for women in areas with low SES compared to high SES was 1.16 (95% CI: 1.02–1.32). Other covariates associated with poorer OS included being 50 years or older, having Medicaid/Medicare insurance, residing in urban areas, being underweight, having higher comorbidities, having a higher stage (II/III) and tumor grade (II/III/IV), undergoing lumpectomy without/unknown radiation, not receiving ODX test, and lacking hormone therapy (Table 3).

Similarly, for BC-specific death, women in areas with low SES had 1.68 times the hazard of death (HR = 1.68; 95% CI: 1.26–2.23) from BC than women in areas with high SES without adjusting other covariates. (Table 4) In the adjusted model, women in areas with low SES had 1.37 times the (HR = 1.37; 95% CI: 1.01–1.87) hazard of dying from BC than women in areas with high SES. Other covariates associated with worse BCSS included being 60 years or older, having a higher stage (II/III), and higher tumor grade (II/III/IV) (Table 4).

### 3.2. Mediation Analysis

Mediation analysis was conducted to quantify the effect of ODX use and stage at diagnosis on the association between SES and OS, whilst adjusting for other covariates or confounders. The total effect (combined effect) of SES on overall survival was 0.27 (95% CI: 0.14, 0.45), indicating that women in areas with low SES had a 30.90% higher hazard of death compared to women in areas with high SES (exp (0.27) = 1.309) (Table 5). The stage at diagnosis significantly explained 11.20% (95% CI: 5.50%, 21.40%) of the SES disparity in OS, with an indirect effect of 0.03 (95% CI: 0.011, 0.049). Additionally, the receipt of the ODX test significantly explained 9.00% (95% CI: 4.30%, 19.30%) of the SES disparity on OS, with an indirect effect of 0.024 (95% CI: 0.006, 0.043). The direct effect of SES on OS, independent of stage and ODX use, was 0.216 (95% CI: 0.091, 0.342), accounting for 79.80% of the total effect.

For BCSS, the total effect of SES was 0.43 (95% CI: 0.13, 0.76), indicating that women in areas with low SES had a 53.70% higher hazard of breast cancer deaths compared to those in areas with high SES (exp (0.43) = 1.537). The stage at diagnosis significantly explained 13.30% (95% CI: 6.50%, 45.20%) of the SES disparities in BC survival, with an indirect effect of 0.058 (95% CI: 0.034, 0.091). Additionally, receipt of the ODX test explained 4.40% (95% CI: 0.9%, 15.9%) of the SES disparities in BC survival, with an indirect effect of 0.019 (95% CI: 0.004, 0.038). The direct effect of SES on BCSS, independent of stage and ODX use, was 0.352 (95% CI: 0.049, 0.671), accounting for 81.50% of the total effect.

## 4. Discussion

In this study, we used mediation analysis to quantify the contributions of ODX testing and stage at diagnosis to the association between SES and OS in women with HR+/HER2- breast cancer, and to the best of our knowledge this is the first such study. We observed that women with low SES had a significantly higher hazard of all causes and BC-specific death, even after adjusting for covariates. We were the first to report that ODX testing explained 9.0% and 4.4% of SES differences in the hazard for OS and BC-specific deaths, respectively. BC stage at diagnosis explained 11.3% and 13.3% of SES differences in the hazard rates for OS and BC-specific deaths, respectively. ODX testing can aid in making informed decisions about chemotherapy by identifying patients at a high risk of recurrence, and therefore, likely to benefit from chemotherapy [12,13]. Moreover, this could allow for the avoidance of unnecessary chemotherapy treatment in patients with a low risk of recurrence [15]. Therefore, ODX testing was associated with improved survival and reduced chemotherapy toxicity, partly explaining its mediating effect on the association of SES with OS [18].

Our study expands on previous research [6,29], which used simpler measures of SES, such as income or education alone, by utilizing Yost’s composite SES measure [30] which includes multiple SES indicators, as mentioned earlier in the introduction. This comprehensive approach provides a more nuanced understanding of the socio-economic disparities affecting BC patient care and outcomes.

The odds of ODX testing were lower for women in areas with low SES compared to high SES women in our study (Table S1), which is consistent with the findings of previous studies [27,28]. Thus, this reflects the impact of socio-economic inequities on the uptake of predictive biomarkers [46] and how ODX testing mediates the association of SES with survival outcome. Specifically, Black women were less likely to receive ODX testing than White women (Table S1), primarily due to lower rates of providers discussion and referral [47,48]. SES plays a crucial role here; most often, individuals of low SES face obstacles in obtaining timely and adequate medical treatment due to lack of or insufficient insurance coverage [49,50,51]. This can lead to delayed detection, resulting in a more advanced and critical stage of cancer, adversely affecting prognosis. This highlights how ODX testing mediates the link between SES and survival rates. Ensuring timely and appropriate cancer care for low-SES women through tests like ODX significantly improves survival rates, advancing equity in healthcare access and outcomes.

Moreover, our data showed that the stage at diagnosis accounted for a significant 11.2% and 13.3% of the variation in overall and BC survival disparities by SES, respectively. This suggests that women from low-SES background were being diagnosed at more advanced stages, which is also clearly demonstrated in Table 1. Our findings were consistent with prior studies, which also found that stage at diagnosis partially explained SES disparities in BC survival [6,52,53]. Generally, earlier detection of BC through screening and improved stage at diagnosis can lead to better survival outcomes [54]. However, SES inequity likely leads to disparities in the utilization of healthcare and screening services among individuals with low SES, resulting in delayed diagnosis and poor outcomes [55]. This may explain how the stage at diagnosis acts as a mediator in SES disparities in BC-specific survival. Additionally, higher stages have a lower probability of BC survival [4,6], further emphasizing the critical role of the stage at diagnosis in mediating the association between SES and survival outcomes. Therefore, enhanced intervention strategies, such as providing free or subsidized medical care to low-income people to prevent delays in cancer diagnosis [50], and increasing screening attendance for women with lower SES [56], can improve survival outcomes in low SES women.

The strength of our study lies in its comprehensive and population-based study, which explores the mediating effect of ODX use and stage on the association of SES and survival among HR+/HER2- BC women. The ODX data were obtained through the NCI-SEER program’s linkage with Genomic Health Inc. (GHI), the only laboratory in the US that performs ODX testing, ensuring the completeness of the data. The mediation effect of ODX testing re-enforces the importance of multigene tests in guiding chemotherapy decisions based on the patient’s recurrence score. Furthermore, the stronger mediation effect of the stage at diagnosis underscores the critical need for targeted interventions to enhance early detection and diagnosis, ultimately improving BC survival outcomes.

Despite several strengths, this study has a few limitations. First, the variables considered in our study do not fully explain SES disparities in BC survival as individual behaviors, detailed healthcare access, and environmental factors influencing survival outcomes were not included. Additionally, using census tract-level SES in our study may not provide an accurate picture of the individual-level SES, which is currently not available in the SEER database. However, it still has robust measures [57] like median household income, median house value, median rent, percent below 150% of the poverty line, education index, percent working class, and percent unemployed.

We also lack data on menopausal status, which can be confounding factor in survival outcomes [58]. However, we adjusted for a strong determinant of age [59] and conducted a study on a specific group, the HR+/HER2- BC women, which helped to mitigate this limitation to a greater extent. Finally, although insurance status may reflect certain dimensions of access to care, it does not capture the full dimension of healthcare utilization and its information uncertainty [60]. Furthermore, the significant disparity in non-receipt of Oncotype DX testing between SES groups highlights the need for equitable access to genomic testing. Future research should investigate the impact of SES on treatment decisions and compliance, as well as explore the biological characteristics of tumors in different SES groups to better understand the factors contributing to disparities in breast cancer outcomes [61]. Moreover, how ODX treatment influences the treatment decision can be explored in future studies. Additionally, the vast majority of patient follow-up is conducted through passive linkages, minimizing the impact of the pandemic. As supporting evidence, the Louisiana Tumor Registry’s follow-up rates continue to meet SEER standards during and post COVID-19 pandemic.

## 5. Conclusions

Among early-stage HR+/HER2- BC women, women with low SES areas had a significantly higher hazard rate of death compared to those with high SES areas. Our findings support the hypothesis that ODX testing and stage at diagnosis mediate the relationship between neighborhood SES and survival. These results highlight the substantial impact of early detection and genomic testing on reducing SES-related survival disparities. Improving access to timely diagnosis and personalized care, particularly for underserved populations, could significantly reduce mortality disparities and improve outcomes in vulnerable groups.

## Figures and Tables

**Table 1:** Early-stage (I–III) HR+/HER2- breast cancer patients and tumor characteristics by socio-economic status (SES) in Louisiana, 2011–2017.

Variable	Total	Low SES	High SES	*p*-Value
	(N = 8931)	(n = 4663, 52.21%)	(n = 4268, 47.79%)	
	n (%)	n (%)	n (%)	
**Race/Ethnicity**				
White	6679 (74.78)	2934 (43.93)	3745 (56.07)	**<0.0001**
Black	2145 (24.02)	1685 (78.55)	460 (21.45)	
Others	107 (1.20)	44 (41.12)	63 (58.88)	
**Age at diagnosis (year)**				
20–49	1328 (14.87)	697 (52.48)	631 (47.52)	0.9255
50–59	2002 (22.42)	1032 (51.55)	970 (48.45)	
60–69	2886 (32.31)	1502 (52.04)	1384 (47.96)	
70–79	1956 (21.90)	1027 (52.51)	929 (47.49)	
80 and over	759 (8.50)	405 (53.36)	354 (46.64)	
**Insurance**				
No insurance	198 (2.22)	138 (69.70)	60 (30.30)	**<0.0001**
Medicaid	974 (10.91)	723 (74.23)	251 (25.77)	
Private	4760 (53.30)	2214 (46.51)	2546 (53.49)	
Medicare	2769 (31.00)	1468 (53.02)	1301 (46.98)	
Other public health insurance ^b^	121 (1.35)	62 (51.24)	59 (48.76)	
Unknown	109 (1.22)	58 (53.21)	51 (46.79)	
**Urban–rural residence**				
Urban (100% urban)	4148 (46.44)	1951 (47.03)	2197 (52.97)	**<0.0001**
Mostly urban (50–100%)	2690 (30.12)	1331 (49.48)	1359 (50.52)	
Mostly rural (0–50%)	1263 (14.14)	713 (56.45)	550 (43.55)	
Rural (100% rural)	830 (9.29)	668 (80.48)	162 (19.52)	
**Body mass index in kg/m^2^**				
Underweight (<18.5)	249 (2.79)	134 (53.82)	115 (46.18)	**<0.0001**
Normal weight (18.5–<25)	1821 (20.39)	775 (42.56)	1046 (57.44)	
Overweight (25–<30)	2351 (26.32)	1215 (51.68)	1136 (48.32)	
Obesity (≥30)	4510 (50.50)	2539 (56.30)	1971 (43.70)	
**Charlson comorbidity scores**				
0	7110 (79.61)	3569 (50.20)	3537 (49.80)	**<0.0001**
1	1372 (15.36)	800 (58.42)	570 (41.58)	
2+	458 (5.13)	294 (64.63)	161 (35.37)	
**AJCC stage**				
I	6186 (69.26)	3125 (50.52)	3061 (49.48)	**<0.0001**
II	2633 (29.48)	1471 (55.87)	1162 (44.13)	
III	112 (1.25)	67 (59.82)	45 (40.18)	
**Tumor grade**				
Grade I	2807 (31.43)	1399 (49.84)	1408 (50.16)	**<0.0001**
Grade II	4390 (49.15)	2262 (51.53)	2128 (48.47)	
Grade III/IV	1488 (16.66)	866 (58.20)	622 (41.80)	
Grade unknown, NR	246 (2.75)	136 (55.28)	110 (44.72)	
**Surgery and reported radiation**				
Lumpectomy plus radiation	4457 (49.90)	2198 (49.32)	2259 (50.68)	**<0.0001**
Mastectomy plus radiation	566 (6.34)	303 (53.53)	263 (46.47)	
Lumpectomy with no/unknown radiation	738 (8.26)	421 (57.05)	317 (42.95)	
Mastectomy with no/unknown radiation	3170 (35.49)	1741 (54.92)	1429 (45.08)	
**Oncotype DX test**				
Yes	3970 (44.45%)	1974 (49.72)	1996 (50.28)	**<0.0001**
No	4961 (55.55%)	2689 (54.20)	2272 (45.80)	
**Chemotherapy received**				
Yes	2140 (23.96)	1184 (55.33)	956 (44.67)	**0.0042**
No	6586 (73.74)	3373 (51.21)	3213 (48.79)	
Unknown	205 (2.30)	106 (51.71)	99 (48.29)	
**Hormone therapy received**				
Yes	6729 (75.34)	3418 (50.80)	3311 (49.20)	**<0.0001**
No	1677 (18.78)	959 (57.19)	718 (42.81)	
Unknown	525 (5.88)	286 (54.48)	239 (45.52)	
**Cause of death**				
Patients alive at last contact	7771 (87.01)	3958 (50.93)	3813 (49.07)	**<0.0001**
BC deaths	207 (2.32)	133 (64.25)	74 (35.75)	
Other cause deaths	953 (10.67)	572 (60.02)	381 (39.98)	

Abbreviations: AJCC = American Joint Committee on Cancer, HR+/HER2- = hormone receptors positive/human epidermal growth factor 2—neu negative, SES = socioeconomic status. Bold indicates statistically significant *p* values. ^b^ Other public health insurance includes Tricare, Military, Veterans Affairs, and Indian/Public Health Service.

**Table 2:** Early-stage (I–III) HR+/HER2- breast cancer patients and tumor characteristics by Oncotype DX (ODX) receipt in Louisiana, 2011–2017.

Variable	ODX Receipt	ODX Not Receipt	*p*-Value
	(n = 3970, 44.45%)	(n = 4961, 55.55%)	
	n (%)	n (%)	
**Socio-economic Status (SES)**			
Group 1 (Low SES)	1974 (42.33)	2689 (57.67)	**<0.0001**
Group 2 (High SES)	1996 (46.77)	2272 (53.23)	
**Race/Ethnicity**			
White	3055 (45.74)	3624 (54.26)	**<0.0001**
Black	861 (40.14)	1284 (59.86)	
Others	54 (50.47)	53 (49.53)	
**Age at diagnosis (year)**			
20–49	659 (49.62)	669 (50.38)	**<0.0001**
50–59	1072 (53.52)	930 (46.45)	
60–69	1463 (50.69)	1423 (49.31)	
70–79	684 (34.97)	1272 (65.03)	
80 and over	92 (12.12)	667 (87.88)	
**Insurance**			
No insurance	82 (41.41)	116 (58.59)	**<0.0001**
Medicaid	389 (39.94)	585 (60.06)	
Private	2368 (49.75)	2392 (50.25)	
Medicare	1024 (40.00)	1745 (63.00)	
Other public health insurance ^b^	68 (56.20)	53 (43.80)	
Unknown	39 (35.78)	70 (64.22)	
**Urban–rural residence**			
Urban (100% urban)	1814 (43.73)	2334 (56.27)	0.1635
Mostly urban (50–100%)	1211 (45.02)	1479 (54.98)	
Mostly rural (0–50%)	591 (46.79)	672 (53.21)	
Rural (100% rural)	354 (42.65)	476 (57.35)	
**Body mass index (BMI) in kg/m^2^**			
Underweight (<18.5)	113 (45.38)	136 (54.62)	0.9873
Normal weight (18.5–<25)	813 (44.66)	1008 (55.35)	
Overweight (25–<30)	1043 (44.36)	1308 (55.64)	
Obesity (≥30)	2001 (44.37)	2509 (55.63)	
**Charlson score**			
0	3259 (45.87)	3847 (54.13)	**<0.0001**
1	553 (40.38)	817 (59.62)	
2+	158 (34.72)	297 (65.28)	
**AJCC stage**			
I	2871 (46.41)	3315 (53.59)	**<0.0001**
II	1088 (41.32)	1545 (58.68)	
III	11 (9.82)	101 (90.18)	
**Tumor grade**			
Grade I, Well-differentiated	1190 (42.39)	1617 (57.61)	**<0.0001**
Grade II, Moderate to Moderately well-differentiated	2073 (47.22)	2317 (52.78)	
Grade III/IV, Poorly differentiated/undifferentiated, anaplastic	624 (41.94)	864 (58.06)	
Grade unknown, NR	83 (33.74)	163 (66.26)	
**Surgery**			
Lumpectomy plus radiation	2194 (49.23)	2263 (50.77)	**<0.0001**
Mastectomy plus radiation	149 (26.33)	417 (73.67)	
Lumpectomy with no/unknown radiation	251 (34.01)	487 (65.99)	
Mastectomy with no/unknown radiation	1376 (43.41)	1794 (56.59)	
**Hormone therapy received**			
Yes	3189 (47.39)	3540 (52.61)	**<0.0001**
No	576 (34.35)	1101 (65.65)	
Unknown	205 (39.05)	320 (60.95)	

Abbreviations: AJCC = American Joint Committee on Cancer, HR+/HER2- = hormone receptors positive/human epidermal growth factor 2—neu negative, SES = socioeconomic status. Bold indicates statistically significant *p* values. ^b^ Other public health insurance includes Tricare, Military, Veterans Affairs, and Indian/Public Health Service.

**Table 3:** Factors associated with overall survival (OS) among early-stage (I–III) HR+/HER2- breast cancer patients in Louisiana, 2011–2017.

Variable	Overall Survival (OS)			
	Crude Model ^c^		Adjusted Model	
	HR	95% CI	HR	95% CI
**Socio-economic Status (SES)**				
Group 1 (Low SES)	**1.45**	**1.29–1.63**	**1.16**	**1.02–1.32**
Group 2 (High SES)	1		1	
**Race/Ethnicity**				
White	1		1	
Black	**1.19**	**1.05–1.35**	0.99	0.86–1.14
Others	0.47	0.21–1.05	0.53	0.24–1.18
**Age at diagnosis (year)**				
20–50 years	1		1	
50–60 years	**1.48**	**1.09–2.02**	**1.72**	**1.26–2.36**
60–70 years	**2.4**	**1.81–3.18**	**2.82**	**2.11–3.78**
70–80 years	**4.88**	**3.70–6.43**	**4.71**	**3.48–6.37**
80–90 years	**10.85**	**8.19–14.38**	**8.63**	**6.29–11.84**
**Insurance**				
No insurance	1.33	0.86–2.06	1.5	0.96–2.33
Medicaid	**2.23**	**1.87–2.66**	**1.53**	**1.27–1.84**
Private	1		1	
Medicare	**2.42**	**2.13–2.75**	**1.19**	**1.03–1.37**
Other public health insurance ^b^	1.12	0.63–1.98	0.9	0.51–1.61
Unknown	1.26	0.67–2.37	1.02	0.54–1.92
**Urban–rural residence**				
Urban (100% urban)	1		1	
Mostly urban (50–100%)	1.06	0.93–1.21	**1.21**	**1.05–1.39**
Mostly rural (0–50%)	1.08	0.91–1.29	1.2	1.00–1.44
Rural (100% rural)	1.22	1.00–1.48	1.11	0.90–1.36
**Body mass index (BMI) in kg/m^2^**				
Underweight (<18.5)	**1.41**	**1.04–1.92**	**1.5**	**1.10–2.04**
Normal weight (18.5–<25)	1		1	
Overweight (25–<30)	**0.8**	**0.67–0.95**	**0.72**	**0.60–0.85**
Obesity (≥30)	0.94	0.81–1.08	**0.84**	**0.72–0.97**
**Charlson score**				
0	1		1	
1	**1.85**	**1.61- 2.13**	**1.49**	**1.29–1.72**
2+	**4.02**	**3.37–4.80**	**2.99**	**2.49–3.59**
**AJCC stage**				
I	1		1	
II	**1.69**	**1.50–1.90**	**1.6**	**1.40–1.82**
III	**3.88**	**2.66–5.68**	**2.58**	**1.72–3.87**
**Tumor grade**				
Grade I, Well-differentiated	1		1	
Grade II, Moderate to Moderately well-differentiated	1.14	0.99–1.32	**1.17**	**1.01–1.35**
Grade III/IV, Poorly differentiated/undifferentiated, anaplastic	**1.74**	**1.48–2.05**	**1.98**	**1.65–2.36**
Grade unknown, NR	**1.64**	**1.20–2.22**	**1.43**	**1.05–1.96**
**Surgery**				
Lumpectomy plus radiation	0.57	0.50–0.65	0.79	0.69–0.90
Mastectomy plus radiation	1.12	0.90–1.40	1.15	0.91–1.46
Lumpectomy with no/unknown radiation	**1.54**	**1.28–1.84**	**1.3**	**1.08–1.56**
Mastectomy with no/unknown radiation	1		1	
**Oncotype DX test**				
Yes	1		1	
No	**2.65**	**2.32–3.03**	**1.67**	**1.45–1.93**
**Chemotherapy received**				
Yes	1		1	
No	**1.22**	**1.06–1.41**	1.12	0.94–1.33
Unknown	0.96	0.62–1.48	0.84	0.53–1.33
**Hormone therapy received**				
Yes	1		1	
No	**1.69**	**1.48–1.93**	**1.28**	**1.12–1.47**
Unknown	**1.38**	**1.09–1.76**	1.18	0.92–1.53

Abbreviations: AJCC = American Joint Committee on Cancer, HR+/HER2- = hormone receptors positive/human epidermal growth factor 2—neu negative, HR = hazard ratios, SES = socioeconomic status. Bold indicates statistically significant values. ^b^ Other public health insurance includes Tricare, Military, Veterans Affairs, and Indian/Public Health Service. ^c^ Crude model refers to the individual association of each covariate with the outcome of interest, analyzed separately without adjustment for other variables.

**Table 4:** Factors associated with breast cancer specific survival (BCSS) among early-stage (I–III) HR+/HER2- breast cancer patients in Louisiana, 2011–2017.

Variable	Breast Cancer—Specific Survival (BCSS)			
	Crude Model ^c^		Adjusted Model	
	HR	95% CI	HR	95% CI
**Socio-economic Status (SES)**				
Group 1 (Low SES)	**1.68**	**1.26–2.23**	**1.37**	**1.01–1.87**
Group 2 (High SES)	1		1	
**Race/Ethnicity**				
White	1		1	
Black	1.33	0.99–1.80	0.99	0.71–1.39
Others	0.9	0.22–3.64	0.76	0.19–3.12
**Age at diagnosis (year)**				
20–50 years	1		1	
50–60 years	0.89	0.54–1.48	1.37	0.82–2.30
60–70 years	1.07	0.68–1.70	**2.03**	**1.24–3.33**
70–80 years	1.66	1.05–2.63	**3.41**	**2.00–5.84**
80–90 years	2.33	1.37–3.96	**4.99**	**2.64–9.45**
**Insurance**				
No insurance	**2.36**	**1.14–4.86**	2.03	0.97–4.26
Medicaid	**1.87**	**1.25–2.81**	1.44	0.94–2.19
Private	1		1	
Medicare	**1.59**	**1.17–2.16**	1.2	0.85–1.71
Other public health insurance ^b^	1.3	0.41–4.11	1.02	0.32–3.27
Unknown	0.57	0.08–4.10	0.71	0.10–5.17
**Urban–rural residence**				
Urban (100% urban)	1		1	
Mostly urban (50–100%)	1.22	0.88–1.69	1.23	0.89–1.71
Mostly rural (0–50%)	1.37	0.92–2.04	1.39	0.92–2.09
Rural (100% rural)	1.44	0.92–2.27	1.17	0.72–1.88
**Body mass index (BMI) in kg/m^2^**				
Underweight (<18.5)	1.47	0.72–3.00	1.51	0.73–3.11
Normal weight (18.5–<25)	1		1	
Overweight (25–<30)	0.81	0.54–1.22	0.69	0.46–1.05
Obesity (≥30)	0.93	0.66–1.32	0.77	0.54–1.11
**Charlson score**				
0	1		1	
1	1.24	0.87–1.78	1.04	0.72–1.50
2+	1.65	0.94–2.90	1.43	0.80–2.56
**AJCC stage**				
I	1		1	
II	3.8	**2.86–5.04**	2.35	**1.72–3.21**
III	10.18	**5.09–20.36**	3.78	**1.78–8.02**
**Tumor grade**				
Grade I, Well-differentiated	1		1	
Grade II, Moderate to Moderately well-differentiated	**2.6**	**1.63–4.15**	**2.19**	**1.37–3.51**
Grade III/IV, Poorly differentiated/undifferentiated, anaplastic	**7.87**	**4.93–12.55**	**5.17**	**3.15–8.49**
Grade unknown, NR	**3.41**	**1.46–7.98**	**2.5**	**1.06–5.90**
**Surgery**				
Lumpectomy plus radiation	**0.69**	**0.51–0.94**	0.95	0.69–1.31
Mastectomy plus radiation	**2.32**	**1.52–3.53**	1.41	0.90–2.21
Lumpectomy with no/unknown radiation	1.26	0.77–2.06	1.48	0.89–2.45
Mastectomy with no/unknown radiation	1		1	
**Oncotype DX test**				
Yes	1		1	
No	**2.02**	**1.50–2.72**	1.29	0.94–1.77
**Chemotherapy received**				
Yes	1		1	
No	**0.34**	**0.26–0.44**	**0.51**	**0.36–0.73**
Unknown	0	-	0	-
**Hormone therapy received**				
Yes	1		1	
No	1.11	0.78–1.57	0.97	0.68–1.40
Unknown	1.02	0.55–1.88	0.91	0.49–1.70

Abbreviations: AJCC = American Joint Committee on Cancer, HR+/HER2- = hormone receptors positive/human epidermal growth factor 2—neu negative, HR = hazard ratios, SES = socioeconomic status. Bold indicates statistically significant values. ^b^ Other public health insurance includes Tricare, Military, Veterans Affairs, and Indian/Public Health Service. ^c^ Crude model refers to the individual association of each covariate with the outcome of interest, analyzed separately without adjustment for other variables.

**Table 5:** Summary of linear mediation effect for overall survival and breast cancer-specific survival.

Mediator	Indirect Effect (95% CI)	Relative Effect (95% CI)
**Overall survival (OS)**		
Stage	0.030 (0.011, 0.049)	**11.20 (5.50, 21.40)**
Oncotype DX	0.024 (0.006, 0.043)	**9.00 (4.30, 19.30)**
Socioeconomic status (Direct effect)	0.216 (0.091, 0.342)	**79.80 (61.80, 88.50)**
Total effect	0.270 (0.144, 0.446)	
**Breast cancer-specific survival (BCSS)**		
Stage	0.058 (0.034, 0.091)	**13.30 (6.50, 45.20)**
Oncotype DX	0.019 (0.004, 0.038)	4.40 (0.90, 15.90)
Socioeconomic status (Direct effect)	0.352 (0.049, 0.671)	**81.50 (41.00, 91.20)**
Total effect	0.432 (0.130, 0.755)	

Note: The mediators are ordered according to the absolute value of the estimated relative effect with linear models. Race, age at diagnosis, insurance, urban–rural residence, body mass index, Charlson comorbidities scores, tumor grade, surgery and reported radiation, chemotherapy, and hormone therapy adjusted in the mediation analysis for overall survival and breast cancer-specific survival. Bold indicates statistically significant values.

## Data Availability

The data supporting the reported results can be found in the Louisiana Tumor Registry. Due to privacy and ethical restrictions, the data are not publicly available. Researchers interested in accessing the data can contact the Louisiana Tumor Registry for further information.

## Supplementary Materials

The following supporting information can be downloaded at: https://www.mdpi.com/article/10.3390/cancers17111802/s1, Table S1: Factors associated with Oncotype DX (ODX) receipt among early-stage (I-III) HR+/HER2- breast cancer patients in Louisiana, 2011–2017.

## List of Abbreviations

Socioeconomic status (SES), Oncotype DX (ODX), breast cancer (BC), overall survival (OS), breast cancer-specific survival (BCSS), hormone receptors positive/human epidermal growth factor 2—neu negative (HR+/HER2-), estrogen receptor (ER), progesterone receptor (PR), Surveillance, Epidemiology, and End Results (SEER), body mass index (BMI), National Comprehensive Cancer Network (NCCN), American Society for Clinical Oncology (ASCO), hazard ratios (HRs), confidence interval (CI), and variance inflation factors (VIF).
