# Supplementary material for: Socioeconomic Disparities in Breast Cancer Survival: Examining Potential Mediator Role of Oncotype DX(ODX) Test and Stage at Diagnosis Among HR+/HER2- Breast Cancer Women
Source: Cancers (Basel). 2025 May 28;17(11):1802. doi: 10.3390/cancers17111802 (PMC12153767; doi:10.3390/cancers17111802)
Supplement: Supplementary file 1 [file cancers-17-01802-s001.zip › cancers-3567630-supplementary.pdf]

cancers

MDPI

Table S1. Factors associated with Oncotype DX (ODX) receipt among early-stage (I-III) HR+/HER2- breast cancer patients in Louisiana, 2011–2017.

Variable	Unadjusted model		Adjusted model	
	OR	95% CI	OR	95% CI
**Socio-economic Status (SES)**				
Group 1 (Low SES)	**0.84**	**0.77 - 0.91**	0.95	0.86 - 1.05
Group 2 (High SES)	1		1	
White	1		1	
Black	**0.80**	**0.72 - 0.88**	**0.79**	**0.70 - 0.88**
Others	1.21	0.83 - 1.77	1.04	0.70 -1.55
**Age at diagnosis (year)**				
20-50 years	1		1	
50-60 years	**1.17**	**1.02 -1.34**	1.08	0.94 - 1.25
60-70 years	1.04	0.91 - 1.19	0.93	0.81 - 1.08
70-80 years	**0.55**	**0.47 - 0.63**	**0.49**	**0.42 - 0.58**
80-90 years	**0.14**	**0.11 - 0.18**	**0.13**	**0.10 - 0.17**
**Insurance**				
No insurance	**0.72**	**0.54 - 0.95**	**0.7**	**0.52 - 0.94**
Medicaid	**0.67**	**0.58 - 0.77**	**0.81**	**0.70 - 0.95**
Private	1		1	
Medicare	**0.59**	**0.54 - 0.65**	0.94	0.84 - 1.06
Other public health insurance [b]	1.3	0.90 - 1.86	1.36	0.93 - 1.99
Unknown	**0.56**	**0.38 - 0.84**	0.7	0.46 - 1.07
**Urban-rural residence**				
Urban (100% urban)	1		1	
Mostly urban (50-100%)	1.05	0.96 - 1.16	0.96	0.87 - 1.07
Mostly rural (0-50%)	1.13	0.99 - 1.29	1.04	0.91 - 1.20
Rural (100% rural)	0.96	0.83 - 1.12	0.94	0.80 - 1.11
**Body mass index (BMI) in kg/m^2**				
Underweight (<18.5)	1.03	0.79 - 1.34	1.13	0.85 - 1.50
Normal weight (18.5-<25)	1		1	
Overweight (25-<30)	0.99	0.87 - 1.12	0.99	0.87 - 1.13
Obesity (≥30)	0.99	0.89 - 1.10	1.01	0.90 - 1.14
**Charlson score**				
0	1		1	
1	**0.80**	**0.71 - 0.90**	0.89	0.80 - 1.02
2+	**0.63**	**0.51 - 0.77**	**0.79**	**0.64 - 0.98**
**AJCC stage**				
I	1		1	
II	**0.81**	**0.74 - 0.89**	**0.88**	**0.80 - 0.98**
III	**0.13**	**0.07 - 0.24**	**0.19**	**0.10 - 0.37**
**Tumor grade**				
Grade I, Well differentiated	1		1	
Grade II, Moderate to Moderately well differentiated	**1.22**	**1.11 - 1.34**	**1.30**	**1.17 - 1.44**
Grade III/IV, Poorly differentiated/undifferentiated, anaplastic	0.98	0.86 - 1.12	1.04	0.91 - 1.20

Grade unknown, NR	**0.70**	**0.53 - 0.91**	0.77	0.58 - 1.02
**Surgery**				
Lumpectomy plus radiation	**1.26**	**1.15 - 1.39**	1.20	1.00 - 1.21
Mastectomy plus radiation	**0.47**	**0.38 - 0.57**	**0.43**	**0.35 - 0.54**
Lumpectomy with no/unknown radiation	**0.67**	**0.57 -0.79**	0.94	0.79 - 1.13
Mastectomy with no/unknown radiation	1		1	
**Hormone therapy received**				
Yes	1		1	
No	**0.71**	**0.59 - 0.85**	**0.66**	**0.59 - 0.75**
Unknown	**0.58**	**0.52 - 0.65**	**0.76**	**0.62 - 0.92**

Abbreviations: AJCC= American Joint Committee on Cancer, HR+/ HER2-=hormone receptors positive/human epidermal growth factor 2 –neu negative, OR=odds ratio. Bold indicates statistically significant values.

[b] Other public health insurance includes Tricare, Military, Veterans Affairs, and Indian/ Public Health Service
